# Supplementary material for: Waiting for the better reward: Comparison of delay of gratification in young children across two cultures
Source: PLoS One. 2021 Sep 3;16(9):e0256966. doi: 10.1371/journal.pone.0256966 (PMC8415579; doi:10.1371/journal.pone.0256966)
Supplement: S1 Table — Generalized linear mixed models (final model) on factors affecting the number of correct test and control trials in children. N = Group 1: China 75; Group 2: UK 61. P-values <0.05 are highlighted in bold. The British dataset was published in Miller et al. [52]. (DOCX) [file pone.0256966.s001.docx]

Waiting for the better reward: Comparison of delay of gratification in young children across two cultures

Ning Ding^1^, Anna Frohnwieser^1^, Rachael Miller*^1 ¶^, Nicola S. Clayton^1¶^

^1^ Department of Psychology, Cambridge University, Cambridge, UK

* Corresponding author

Email: [rmam3@cam.ac.uk](mailto:rmam3@cam.ac.uk) (RM)

^¶^ = these authors contributed equally to this work (joint senior authorship)

**Experiment 1 (**Test and control trials combined**)**

In the test and control trials, the full models differed significantly from the null models

(Chisq = 149.46, df = 8, *p* = <0.001). In Experiment 1, the full model was not significantly different to the reduced model i.e. main effects only (Chisq = 2.533, df = 2, p=0.282). Therefore the interaction term (Age: Country) did not significantly improve the model and the final reduced model reported is the best fit (S1 Table). We found a significant main effect of **condition** (quality vs quantity), **age** (3-5 years) and **trial type** (test vs control) (S1 Table).

**S1 Table. Generalized linear mixed models for Experiment 1.**

| **Fixed term** | **Chi-square** | **df** | **p-value** |
| --- | --- | --- | --- |
| Country | 0.6445 | 1 | 0.4221 |
| **Trial type** | 1.795 | 10.606 | **<0.0001** |
| **Condition** | 12.846 | 1 | **<0.001** |
| **Age in years** | 8.867 | 2 | **0.012** |
| Order | 2.262 | 1 | 0.133 |
| Sex | 0.169 | 1 | 0.681 |

Generalized linear mixed models (final model) on factors affecting the number of correct test and control trials in children. N = Group 1: China 75; Group 2: UK 61. P-values <0.05 are highlighted in bold. The British dataset was previously published in Miller et al. (53).
