# Supplementary material for: Waiting for the better reward: Comparison of delay of gratification in young children across two cultures
Source: PLoS One. 2021 Sep 3;16(9):e0256966. doi: 10.1371/journal.pone.0256966 (PMC8415579; doi:10.1371/journal.pone.0256966)
Supplement: S2 Table — Generalized linear mixed models (final model) on factors affecting the number of correct test and control trials in children. N = Group 1: China 75; Group 2: UK 61. P-values <0.05 are highlighted in bold. The British dataset was published in Miller et al. [52]. (DOCX) [file pone.0256966.s002.docx]

Waiting for the better reward: Comparison of delay of gratification in young children across two cultures

Ning Ding^1^, Anna Frohnwieser^1^, Rachael Miller*^1 ¶^, Nicola S. Clayton^1¶^

^1^ Department of Psychology, Cambridge University, Cambridge, UK

* Corresponding author

Email: [rmam3@cam.ac.uk](mailto:rmam3@cam.ac.uk) (RM)

^¶^ = these authors contributed equally to this work (joint senior authorship)

**Experiment 2** (Test and control trials combined)

In the test and control trials, the full models differed significantly from the null models (Chiq = 206.95, df = 10, *p* = <0.001). In Experiment 2, the full model was significantly different to the reduced model i.e. main effects only (Chiq = 151.9, df = 3, p=<0.001). Therefore the interaction term (Age: Country) significantly improved the model and the final reduced model reported is the best fit (S1 Table). We found a significant main effect of **condition** (quality vs quantity), and **trial type** (test vs control), with a significant interaction effect of **country: age** (UK vs China: 3 to 5 years) (S2 Table).

**S2 Table. Generalized linear mixed models for Experiment 2.**

| **Fixed term** | **Estimate** | **z-value** | **p-value** |
| --- | --- | --- | --- |
| **Trial type** | 137.27 | 1 | **<0.001** |
| **Condition** | 17.613 | 1 | **<0.001** |
| Order | 0.01 | 1 | 0.919 |
| Sex | 0.069 | 1 | 0.705 |
| Visibility | 1.907 | 2 | 0.386 |
| **Country: Age** | 54.97 | 5 | **<0.001** |

Generalized linear mixed models (final model) on factors affecting the number of correct test and control trials in children. N = Group 1: China 75; Group 2: UK 61. P-values <0.05 are highlighted in bold. The. British dataset was previously published in Miller et al. (53).
