# Supplementary material for: Waiting for the better reward: Comparison of delay of gratification in young children across two cultures
Source: PLoS One. 2021 Sep 3;16(9):e0256966. doi: 10.1371/journal.pone.0256966 (PMC8415579; doi:10.1371/journal.pone.0256966)
Supplement: S3 Table — Generalized linear mixed models (final model) on factors affecting the number of correct test trials in children for Experiment 2 with the 3 British 4-year-old outliers removed. N = Britain 58; China 75. P-values <0.05 are highlighted in bold. The British dataset was published in Miller et al. [52]. (DOCX) [file pone.0256966.s003.docx]

Waiting for the better reward: Comparison of delay of gratification in young children across two cultures

Ning Ding^1^, Anna Frohnwieser^1^, Rachael Miller*^1 ¶^, Nicola S. Clayton^1¶^

^1^ Department of Psychology, Cambridge University, Cambridge, UK

* Corresponding author

Email: [rmam3@cam.ac.uk](mailto:rmam3@cam.ac.uk) (RM)

^¶^ = these authors contributed equally to this work (joint senior authorship)

**Experiment 2 with dataset without outliers**

Experiment 2 post-hoc comparisons between British and Chinese children without outliers

As with data set including outliers, Chinese 4- and 5-year-old children outperformed their British peers, with no difference in performance between the Chinese and British 3-year-olds, when outliers were removed for analysis.

**S3 Table. Generalized linear mixed models for Experiment 2 without outliers.**

| Fixed Term | Chi-square | df | p-value |
| --- | --- | --- | --- |
| Condition | 16.83 | 1 | **<0.001** |
| Country: Age | 39.79 | 5 | **<0.001** |
| Order | 0.098 | 1 | 0.755 |
| Sex | 0.232 | 1 | 0.526 |
| Visibility | 3.159 | 2 | 0.206 |

Generalized linear mixed models (final model) on factors affecting the number of correct *test* trials in children for Experiment 2 with the 3 British 4-year-old outliers removed. N = Britain 58; China 75. P-values <0.05 are highlighted in bold. The British dataset was previously published in Miller et al. (53).
