# Supplementary material for: Waiting for the better reward: Comparison of delay of gratification in young children across two cultures
Source: PLoS One. 2021 Sep 3;16(9):e0256966. doi: 10.1371/journal.pone.0256966 (PMC8415579; doi:10.1371/journal.pone.0256966)
Supplement: Attachment — Submitted filename: Response to Reviewers - 2nd revision.docx [file pone.0256966.s006.docx]

Dear Dr Addessi,

Many thanks for inviting us to revise our manuscript [PONE-D-21-06290R1] entitled “Waiting for the Better Reward: Comparison of Delay of Gratification in Young Children across Two Cultures”.

We wish to thank you and the two reviewers for helpful feedback. We have now addressed the comments. Please find responses to each comment below. Please note that line numbers correspond with the tracked changes version of the manuscript.

We hope that following our revisions, you will consider our manuscript for publication in PLoS ONE.

Yours Sincerely.

Ning Ding, Anna Frohnwieser, Rachael Miller, and Nicky Clayton

**Response to Editor and Reviewers:**

**Editor comment:**

In particular, please carefully address the issues raised by Reviewer 2 concerning **statistical analyses** and report also in the **abstract, introduction, results and discussion** that the data on **British children** have already been collected for a previous study.

**We have now addressed these comments in the ms and in response to reviewers below.**

**Reviewer comments:**

**Reviewer #1:** I appreciate the authors' responsiveness to the reviewer feedback and believe that they have adequately addressed the comments from the previous round for an improved and interesting manuscript.

**Thank you very much.**

As a very minor comment, when referencing the studies (47-52), on lines 153-154 and 702-704, be sure to be inclusive of the pig reference (right now, the authors refer to these studies as primates and avian species but study 51 includes pigs).

**We have changed the text to state that the research includes primates, pigs, and avian species (line 141-142, line 703).**

**Reviewer #2:** The authors have considerably revised their manuscript and I found the overall manuscript improved. The authors have addressed the majority of reviewer comments, however, there remain a few issues that I think would need to be addressed and/or clarified in a further revision.

**Thank you very much, we have now addressed these additional comments.**

(1) Use of previously collected data:
I commented in my initial review that the fact that the British data originates from a previously published study did not become clear until the methods section and recommended to centre the paper more on the novel Chinese data. The authors responded that the comparison of the two data sets is central to their paper and that they did not want to emphasize one group over the other. I have no objections to this point (it is up to the authors to decide on the emphasis of their paper); however, the authors still do not clearly state in the abstract or in the introduction that the only novel data are the Chinese data. In fact, statements in the abstract like “Here, we tested delay of gratification in 136 3 to 5-year-old British (n=61) and Chinese (n=75) children using Bramlett et al. (1) delay choice paradigm…” give the impression that all the presented data were novel. For transparency reasons, I think it is important to clearly state throughout the manuscript that the British data used for comparison has previously been published (and reference the respective publication). I think this needs to be stated (and the publication referenced) throughout including the abstract, introduction, figure captions, table headings and discussion section, so even readers how may skip sections of the paper are aware of this.

**Thank you for pointing out the need to state the use of previously collected data and it is now explicitly stated in the Abstract (line 37), Introduction (line 233-235), figure captions (line 531-532, line 538, line 588), table legends (line 527, line 582) and discussion (line 633-635).**

(2) Statistical analyses:
In my previous comments, I asked the authors for some clarifications about their statistical approach. They have now provided further details and helpfully provided examples from their Rscript (their data is also available for download from figshare). However, I have some remaining comments/questions about the analyses.

(2a) **P-values** of main effects in the best fit model: Unfortunately, based on the Rscript provided it still did not fully become clear how the p-values were determined. It seems like the authors manually removed each factor of interest and then compared models, but the full script is not provided (only what seems like an example is provided). [Note: likelihood ratio tests for all factors can be quickly done with the drop1() function, using the best-fit model as input and setting the test statistic to chi-square; e.g. drop1(m4, test=”Chisq”).]

On my second reading of the paper, I also noticed that the model tables (Table 1 and 2) report **z-values for the estimates.** If the reported p-values are derived from likelihood ratio tests, then the reported test statistic would need to be **chi-square values and dfs** from those tests. The z-values refer to approximate p-values from the model outputs and do not correspond to the likelihood ratio test statistic (which is a chi-square test statistic).

**We hope we have improved clarity of the R script (**<https://figshare.com/s/01356d6162a8b55137c4>**) and updated the GLMM results in the manuscript and supplementary materials to include chi-square values and dfs rather than z-values for estimates (Table 1, Table 2, S1, S2, S3). The p-values have been checked and updated if required and reflect those obtained from the likelihood ratio tests.**

(2b) Age as continuous vs. categorical variable in the GLMMs: The authors now clarified that age was entered as categorical variable in the GLMMs. However, the R-script does not **convert age into a factor before analysis** and age is a numerical variable in the data file. So based on this information, it seems that age may have been entered as a continuous variable? Could the authors double check and, if necessary, re-run their analyses with age as categorical variable?

**We have re-run our analyses ensuring that age was converted into a factor before analysis (i.e. as a categorical variable). There are no changes for Experiment 1 (Fig 4 updated to better represent the overall age effect, line 534-538). In Experiment 2, age and country are no longer significant main effects, while the country: age interaction and condition remains significant. We have updated the results section accordingly (Table 2, line 558-559, line 564-577) and modified Fig 5 (line 584-588) to present the interaction effect. We also changed the supplementary tables (outliers removed) and confirm again that there are no significant changes to the results from Experiment 2 when the outliers are removed.**

(2c) Additional non-parametric post-hoc tests: I commented in my previous review that I am puzzled about the non-parametric follow-up tests and the authors offered two reasons for conducting them (a) “to test the direction of these effects” and (b) “comparable analysis with our previous related study (Miller et al., 2019, Animal Cognition”.
About (a): the model estimates (i.e., their sign) provide information about the direction of the effects; for example, the estimate of condition in experiment 1 is negative and assuming that quality was set as reference level, this reveals that children’s success rate was lower in the quantity as compared to the quality condition. For any factor with two levels (which are the majority of the authors’ factors), the initial model output is sufficient to interpret the direction of the effects. For factors with more than 2 levels (such as age), the authors could set the reference level of the factor to e.g. 3yos using the relevel() function and will then get estimates for 3 vs. 4 and 3 vs. 5; if they also want an estimate of 4 vs. 5 they can change the reference level to 4yos and re-run the model.
About (b): I had a look at the methods and results section of the previous paper (Miller et al., 2019). The paper reports model comparisons and tables with model estimates and p-values (derived from, what I assume, are likelihood ratio tests). Non-parametric tests were only reported for comparisons against chance; no non-parametric test statistics for e.g. comparing the two conditions were reported. So, it seems that the authors directly interpreted the model estimates in their previous article (?) and only used non-parametric tests for comparisons against chance. (Note that my puzzlement only concerns the use of non-parametric effects to interpret the model effects, not their use for comparisons against chance.)

**We have followed the reviewer’s suggestion and now directly interpret the model estimates rather than reporting non-parametric test results for within-factor comparisons (e.g. sex, country, order, condition). We also now use Tukey contrasts to report pair-wise comparisons within the (3-level) factor of age where applicable and updated the manuscript accordingly (line 542-545; line 566-569; line 572-575). We continue to use non-parametric tests to report comparisons against chance, which the reviewer supported in their comment.**

(3) Discussion, p. 21, line 668 ff.: This paragraph makes some broad claims about country-level differences in performance although the data is more nuanced than that. In fact, the authors have a very insightful and nuanced discussion of cultural similarities and differences later in the discussion section and I think the later section does better justice to their data. So, I would recommend removing the paragraph in lines 668-678.

**We are very glad that the reviewer considered our discussion insightful and nuanced. We have included the content about societal difference between the Eastern and Western as a broader influence of children’s cognitive development and it also echoes the literature we summarised in the introduction. We think it’s worth keeping it because it might help with readers who haven’t read the introduction or with limited knowledge of cross-cultural research.**

Minor comment:
The authors frequently use wording like "Bramlett et al. (1) delay choice paradigm" when referencing paradigms or previous findings. I think this may need to be changed to "Bramlett et al.'s (1) delay choice paradigm" etc..

**We have changed the reference throughout the manuscript.**
